# Incidence and clinicopathological features of colorectal cancer among multi-ethnic patients in Kuala Lumpur, Malaysia: a hospital-based retrospective analysis over two decades

**DOI:** 10.7717/peerj.12425

**Published:** 2021-11-08

**Authors:** Khairul Najmi Muhammad Nawawi, Norfilza M. Mokhtar, Zhiqin Wong, Zairul Azwan Mohd Azman, Deborah Chia Hsin Chew, Rasyidah Rehir, Jocelyn Leong, Fuad Ismail, Isa Mohamed Rose, Yazmin Yaacob, Hamzaini Abdul Hamid, Ismail Sagap, Raja Affendi Raja Ali

**Affiliations:** 1 Gastroenterology Unit, Department of Medicine, Faculty of Medicine, Universiti Kebangsaan Malaysia, Cheras, Kuala Lumpur, Malaysia; 2 GUT Research Group, Faculty of Medicine, Universiti Kebangsaan Malaysia, Cheras, Kuala Lumpur, Malaysia; 3 Department of Physiology, Faculty of Medicine, Universiti Kebangsaan Malaysia, Cheras, Kuala Lumpur, Malaysia; 4 Colorectal Unit, Department of Surgery, Faculty of Medicine, Universiti Kebangsaan Malaysia, Cheras, Kuala Lumpur, Malaysia; 5 Department of Oncology, Faculty of Medicine, Universiti Kebangsaan Malaysia, Cheras, Kuala Lumpur, Malaysia; 6 Department of Pathology, Faculty of Medicine, Universiti Kebangsaan Malaysia, Cheras, Kuala Lumpur, Malaysia; 7 Department of Radiology, Faculty of Medicine, Universiti Kebangsaan Malaysia, Cheras, Kuala Lumpur, Malaysia

**Keywords:** Colorectal cancer, Incidence, Malaysia, Multi-ethnic, EOCRC, Early-onset, Kuala Lumpur

## Abstract

**Background:**

The incidence rate of colorectal cancer (CRC) in Asian countries is increasing. Furthermore, recent studies have shown a concerning rise in the incidence of CRC among younger patients aged less than 50 years. This study aimed to analyze the incidence trends and clinicopathological features in patients with early-onset CRC (EOCRC) and later-onset CRC (at age ≥ 50 years).

**Methods:**

A retrospective analysis was performed on 946 patients with CRC diagnosed from 1997 to 2017 at Universiti Kebangsaan Malaysia Medical Centre. The time trend was assessed by dividing the two decades into four 5-year periods. The mean age-standardized and age-specific incidence rates were calculated by using the 5-year cumulative population of Kuala Lumpur and World Health Organization standard population. The mean incidence was expressed per 100,000 person-years.

**Results:**

After a stable (all age groups) CRC incidence rate during the first decade (3.00 per 100,000 and 3.85 per 100,000), it sharply increased to 6.12 per 100,000 in the 2008–2012 period before decreasing to 4.54 per 100,000 in the 2013–2017 period. The CRC incidence trend in later-onset CRC showed a decrease in the 2013–2017 period. Contrariwise, for age groups of 40–44 and 45–49 years, the trends showed an increase in the latter 15 years of the study period (40–44 years: 1.44 to 1.92 to 2.3 per 100,000; 45–49 years: 2.87 to 2.94 to 4.01 per 100,000). Malays’ EOCRC incidence rate increased from 2008–2012 to 2013–2017 for both the age groups 40–44 years (1.46 to 2.89 per 100,000) and 45–49 years (2.73 to 6.51 per 100,000). Nearly one-fifth of EOCRC cases were diagnosed at an advanced stage (Dukes D: 19.9%), and the majority of them had rectal cancer (72.8%).

**Conclusion:**

The incidence of EOCRC increased over the period 1997–2017; the patients were predominantly Malays, diagnosed at a later stage, and with cancer commonly localized in the rectal region. All the relevant stakeholders need to work on the management and prevention of CRC in Malaysia.

## Introduction

Colorectal cancer (CRC) is the third most common cancer worldwide and has become the second-leading cause of cancer death in the world. Its global incidence varies across regions (Global Burden of Disease Cancer Collaboration et al., 2017; Arnold et al., 2017). CRC rates are rising in the Asia-Pacific region and have become a major public health concern, with Malaysia being no exception (Center, Jemal & Ward, 2009). Recent publications have drawn attention towards early-onset CRC (EOCRC), for which there has been an increasing incidence trend globally (Siegel et al., 2019; Abualkhair et al., 2020). EOCRC is defined as CRC in a patient aged less than 50 years. This cut-off point was derived from many studies that showed an increased risk of CRC after the fifth decade of one’s life.

Malaysia is a developing country located in Southeast Asia. It had an estimated population of 32.4 million in 2018, with an annual population growth rate of 1.1%. Malaysia is blessed with diverse ethnic groups, with three major groups—namely, Malay (69.1%), Chinese (23%), and Indian (6.9%) ethnic groups—constituting the Malaysian population (Department of Statistic Malaysia, 2017). CRC is the most common cancer in males (16.9% of all cancers diagnosed) and the second most common cancer in females (10.7% of all cancers diagnosed) according to the Malaysian National Cancer Registry Report 2012–2016 (Azizah et al., 2019). Another Malaysian study looking at the National Cancer Registry for CRC from 2008 to 2013 revealed that the overall age-standardized incidence rate for CRC was 21.32 per 100,000 population. Those of Chinese ethnicity had the highest CRC incidence rate (27.35), followed by Malay (18.95) and Indian (17.55) (Abu Hassan et al., 2016).

The economic burden of CRC in Malaysia is substantial and is expected to continue to rise. A study by Ezat et al. indicated that the mean cost (direct and indirect) of CRC treatment per person per year was MYR 13,622 for stage 1, MYR 19,752 for stage 2, MYR 24,972 for stage 3, and MYR 27,377 for stage 4, with the total management cost of all new CRC cases estimated to be around MYR 108 million (Natrah et al., 2012; Veettil et al., 2017). Despite the significant disease and economic burdens implicated by CRC, local studies pertaining to this field are scarce. Hence, we aimed to analyze the time trend, clinicopathological features, and treatment modalities received by CRC patients at our single tertiary care center, Universiti Kebangsaan Malaysia Medical Centre (UKMMC) in Kuala Lumpur, from 1997 to 2017.

## Materials and Methods

### Study setting, design, and data collection

All CRC patients diagnosed and treated at UKMMC from 1997 to 2017 were included in this retrospective cohort study. The CRC cases were captured from all relevant subspecialty teams in the hospital, including the medical, surgical, and oncology units. Data were collected primarily from the hospital medical record systems, which comprised the patients’ medical files and online patient information database. This study was approved by the Research Ethics Committee, Universiti Kebangsaan Malaysia (Ethics Committee/IRB Reference No: UKM PPI/111/8/JEP-2017-762), and was performed in accordance with the ethical standards laid down in the 1964 Declaration of Helsinki and its later amendments. Written consents were obtained from the patients and/or their family members (in the case of deceased patients).

UKMMC is a university teaching hospital in Malaysia. It is a tertiary care center located in Cheras, Kuala Lumpur. It was founded in 1997, and the hospital services an urban multi-ethnic population in Kuala Lumpur. It has over 1000 beds across all subspecialties, with an average of 36,000 admissions per year. The total population in Kuala Lumpur in 2017 was 1,793,200 people (5.6% of Malaysia’s population), and 86.3% of them were Malaysian citizens. Regarding the ethnic groups in Kuala Lumpur, the majority of inhabitants are Malay (46.8%), followed by Chinese (42.2%) and Indian (9.8%) ethnicity (Department of Statistic Malaysia, 2017).

### Parameter definitions

A total of 946 patients had pathologically confirmed CRC and were included in the analysis. The three major ethnic groups that were included in this study were Malay, Chinese, and Indian. The other included ethnic groups were Punjabis, Bidayuh, and Kadazan, among which all were long-term residents of Kuala Lumpur. The main analysis was performed by comparing two CRC groups, namely, EOCRC (patients aged between 18 and 49 years) and later-onset CRC (patients aged 50 years and above). Furthermore, a sub-analysis of time trends of CRC incidence rates in the age groups 40–44 and 45–49 years was also performed, since the incidence rates of EOCRC were the highest in these age groups.

Proximal CRC was defined as tumors located at the transverse colon, hepatic flexure, ascending colon, or caecum. On the other hand, distal CRC was defined as tumors located at the splenic flexure, descending colon, sigmoid colon, rectum, or anus.

### Incidence rate of CRC

Time trend analysis of CRC incidence at UKMMC was illustrated by comparing the mean incidence rates of CRC in every 5-year period (1997–2002, 2003–2007, 2008–2012, and 2013–2017). The mean age-standardized and age-specific incidence rates were calculated by using the 5-year cumulative population of Kuala Lumpur and World Health Organization standard population. The mean incidence was expressed per 100,000 person-years.

### Statistical analysis

Continuous variables were calculated by using the mean and standard deviation, while categorical variables were summarized as proportions and percentages. The Chi-squared test was used to compare differences between categorical variables; a two-tailed significance level of 0.05 was applied. Unknown or missing data were recorded as not reported in the analysis. All the data were analyzed using IBM SPSS Statistics version 24.0 (IBM Corporation, New York, USA).

## Results

### Time trends of CRC incidence rates, all age groups

**Figure 1:**
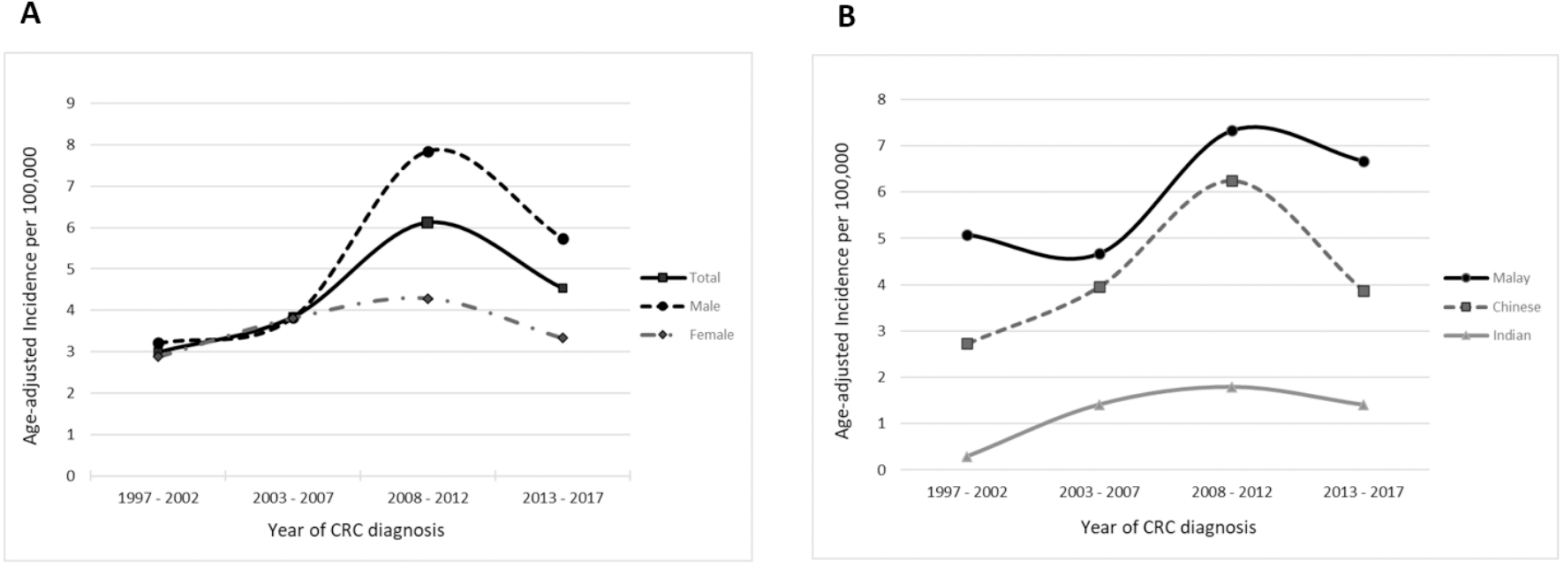
Time trends for the age-adjusted total incidence rate of CRC stratified by (A) gender and (B) ethnicity.

The total incidence rates of CRC cases for the first decade were stable at 3.00 and 3.85 per 100,000 person-years for the 1997–2002 and 2003–2007 periods, respectively. In the first half of the second decade (2008–2012), the incidence rate sharply increased (6.12 per 100,00 person-years), before decreasing to 4.54 per 100,000 person-years towards the end of the second decade (2013–2017). When stratified according to gender, the previously seen time trend in the total CRC incidence rate was mainly contributed by an increased incidence of male CRC. Female CRC, on the other hand, showed a relatively constant incidence rate throughout the two decades (Table 1, Fig. 1A).

**Table 1:** Age-adjusted incidence rates of colorectal cancer.

	Age-adjusted incidence rate (per 100,000 person-years)					
Time period	Total	Male	Female	Malay	Chinese	Indian
1997–2002 2003–2007 2008–2012 2013–2017	3.00 3.85 6.12 4.54	3.21 3.83 7.84 5.74	2.88 3.81 4.28 3.33	5.08 4.67 7.33 6.67	2.73 3.96 6.25 3.87	0.29 1.41 1.79 1.40

Consistently, over the two decades, those of Malay ethnicity had the highest incidence rates, followed by those of Chinese ethnicity. Those of Indian ethnicity presented a steadily low incidence rate throughout the two decades (range: 0.29 to 1.79 per 100,000 person-years). When examining the CRC trends in the Malay and Chinese subgroups, the incidence rates for both increased significantly in the 2008–2012 period as compared to the 2003–2007 period (Malay: from 4.67 to 7.33 per 100,000 person-years; Chinese: from 3.96 to 6.25 per 100,000 person-years). In the 2013–2017 period, the Chinese subgroup experienced a considerable decrease in incidence rate (−38%), as compared to a slight decrease in the Malay subgroup incidence rate (−9%) (Fig. 1B).

### Time trends of CRC incidence rates, per age group

**Figure 2:**
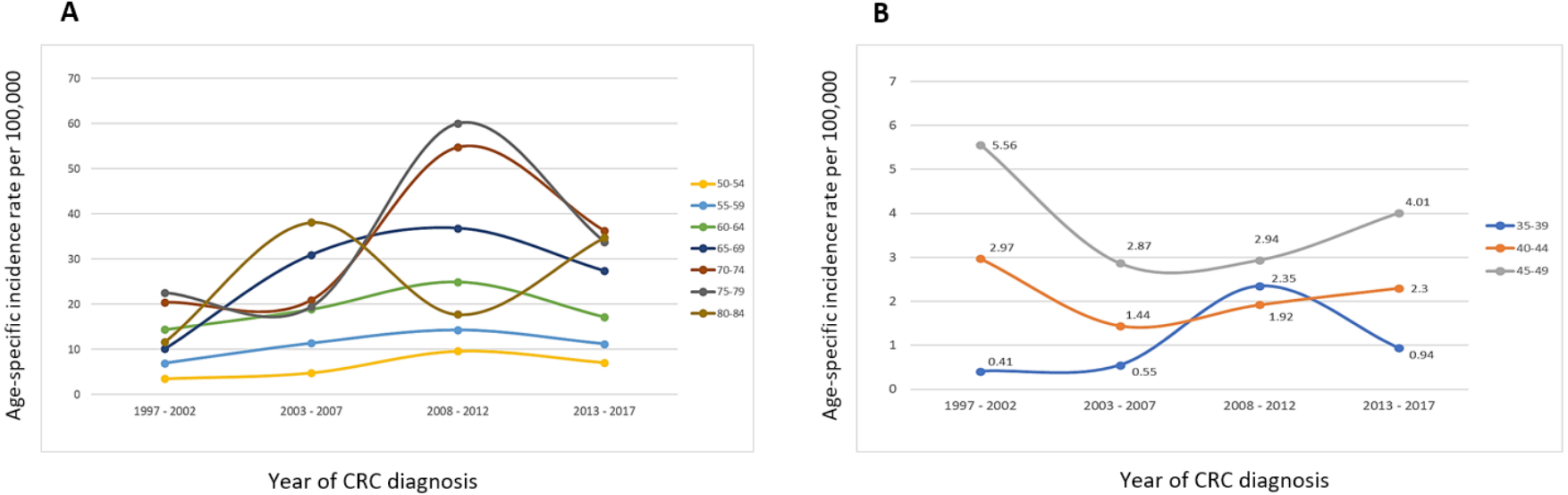
Time trends for the age-specific incidence rate of total CRC stratified by age groups (A) 50–84 years and (B) 35–49 years.

Analysis of the time trends of the CRC incidence rates for age groups 50 years and above revealed a decreasing trend from 2008–2012 to 2013–2017, except for the age group 80–84 years. Contrariwise, in the younger age groups (40–44 and 45–49 years), the incidence rates increased steadily from 2003–2007 until 2013–2017 (Fig. 2).

### Sub-analysis of age groups 40–44 and 45–49 years: ethnicity, Dukes stage, and CRC location

**Figure 3:**
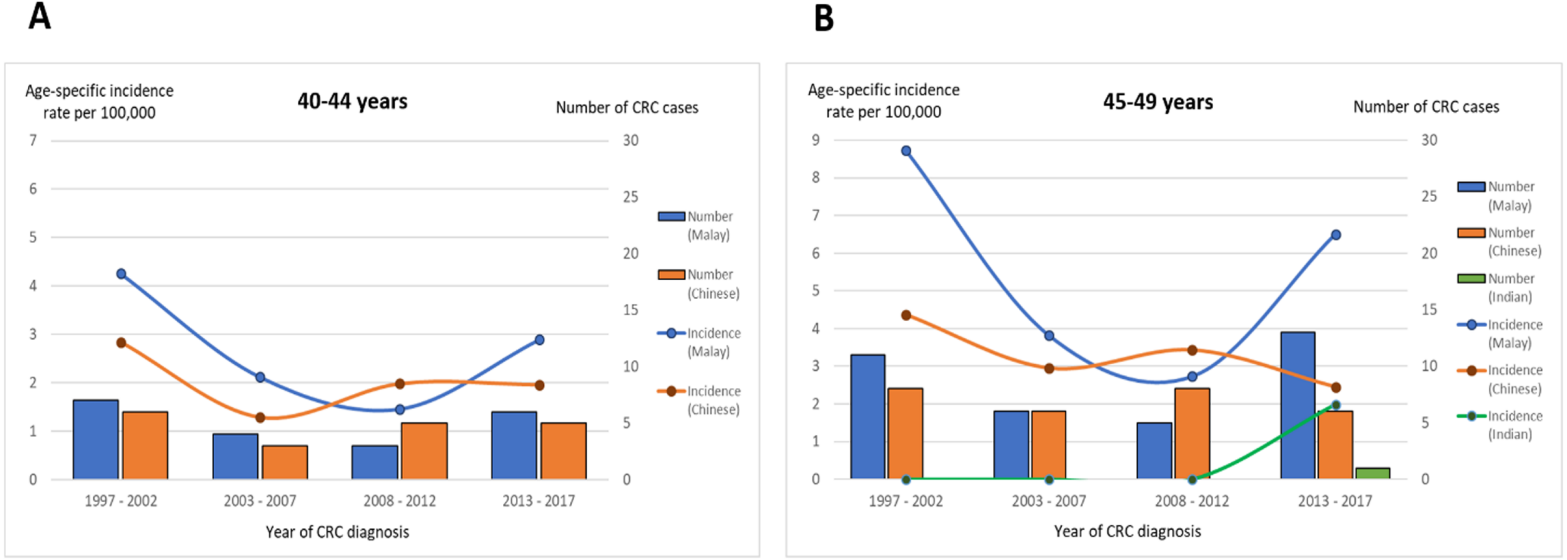
Time trend for age-specific incidence rate and number of CRC cases stratified by ethnicity and age groups (A) 40–44 years and (B) 45–49 years.

The time trends of the CRC incidence rates in the Malay subgroup over the two decades were similar for the age groups 40–44 and 45–49 years. The incidence rates decreased initially from the first until the third time period, before starting to increase in the 2013–2017 period (40–44 years: 4.3 per 100,000 person-years in 1997–2002, 2.1 per 100,000 person-years in 2003–2007, 1.5 per 100,000 person-years in 2008–2012; 45–49 years: 8.7 per 100,000 person-years in 1997–2002, 3.8 per 100,000 person-years in 2003–2007, 2.7 per 100,000 person-years in 2008–2012). Notably, in the age group 45–49 years, there was an enormous increase of 138% in the Malay subgroup’s CRC incidence rate in the 2013–2017 period from that in the 2008–2012 period (6.51 from 2.73 per 100,000 person-years). On the other hand, there was no significant variation in the time trend of the Chinese subgroup’s CRC rate over the two decades; the latest CRC incidence rates were either constant (40-44 years: 1.96 from 1.98 per 100,000 person-years) or minimally decreasing (45–49 years: 2.45 from 3.43 per 100,000 person-years) (Fig. 3).

**Figure 4:**
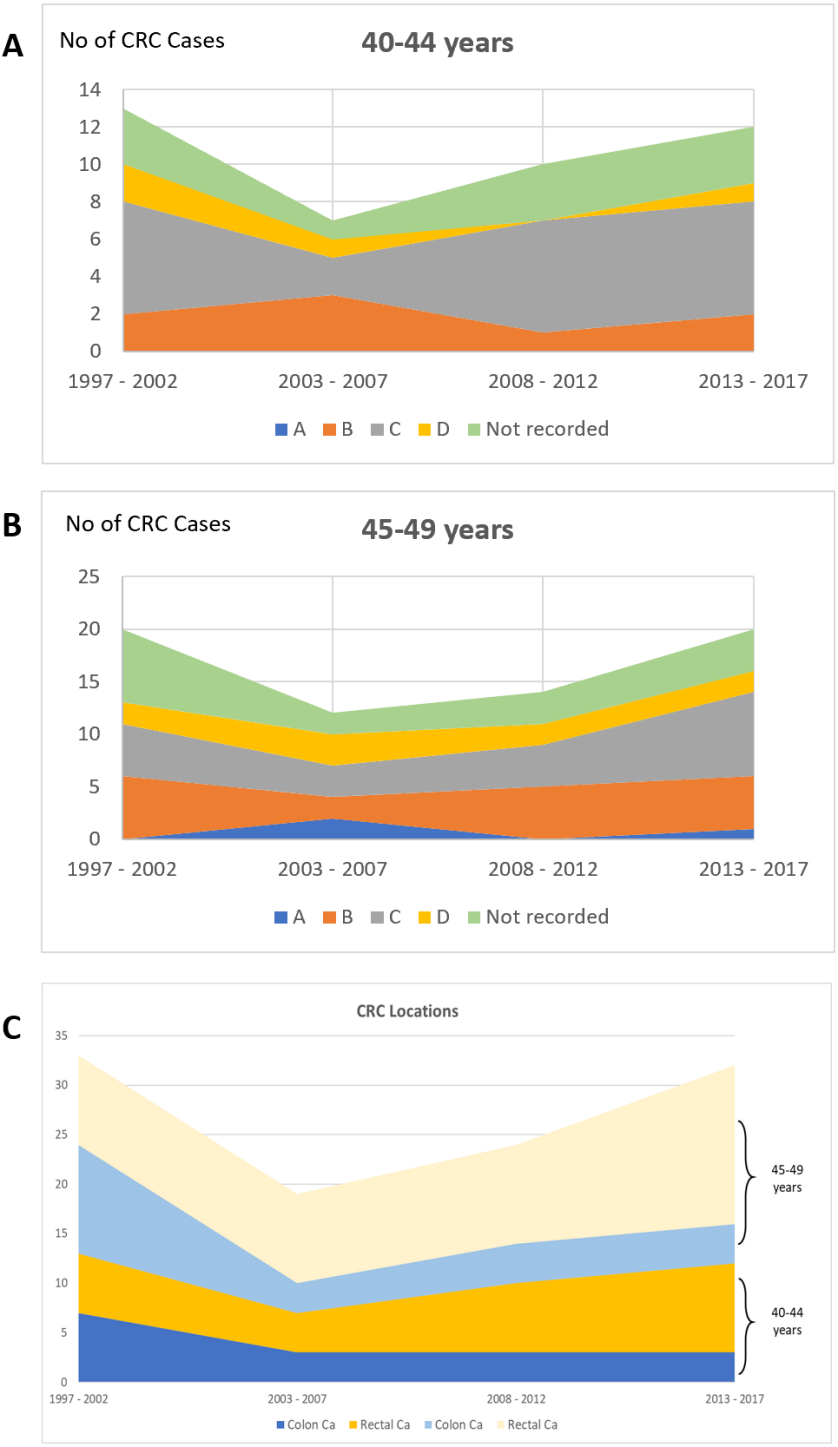
Proportion of CRC patients over the four time periods in age groups of 40–45 and 45–49 years according to (A) & (B) Dukes’ stage and (C) CRC location.

Considering the Dukes stage for EOCRC in the age groups 40–44 and 45–49 years, the majority were diagnosed at Dukes B and C (40–44 years: 7 patients at Dukes B and 20 patients at Dukes C; 45–49 years: 18 patients at Dukes B and 20 patients at Dukes C). Regarding CRC location, the rectum was the most common location throughout the four 5-year periods in both age groups, more frequent than the colon (40–44 years: 23 patients with rectal cancer *vs.* 16 patients with colon cancer; 45–49 years: 41 with rectal cancer *vs.* 20 patients with colon cancer) (Fig. 4).

### Demographic characteristics of CRC patients: EOCRC *vs.*≥50 years

Overall, the mean age at diagnosis was 41.1 years (±7.1) for EOCRC and 61.3 years (±11.9) for those aged ≥50 years. There was a slight male predominance (1.5:1) of CRC in both age groups. With regards to ethnicity, the Malay subgroup had the highest number of CRC cases in the EOCRC group (*n* = 91, 57.2%), while more CRC cases were found in the Chinese subgroup for the age group ≥50 years (*n* = 437, 55.5%). The number of CRC cases was very low among Indian patients (1.9% *vs.* 3.7% among EOCRC and those aged ≥50 years, respectively). Out of 159 patients with EOCRC, only 3 of them had a known genetic risk of CRC (2 had familial adenomatous polyposis (FAP) and 1 had Lynch Syndrome) and only 1 had a first-degree family history of CRC. Looking at the age group ≥50 years, 15 patients (1.9%) had a family history of CRC, among which 2 patients had a family history of Lynch Syndrome (Table 2).

**Table 2:** Demographic details and treatment modalities of the two main examined age groups of colorectal cancer patients.

Patient characteristics	Age <50 years, [total:159]	Age ≥50 years, [total:841]	*P* value
Age, years, mean (standard deviation)	41.1 (7.1)	65.0 (8.3)	
**Age group; years, n (%)**			
15–19	1 (0.6)	–	
20–24	5 (3.2)	–	
25–29	7 (4.4)	–	
30–34	14 (8.8)	–	
35–39	24 (15.1)	–	
40–44	42 (26.4)	–	
45–49	66 (41.5)	–	
50–54	–	101 (12.0)	
55–59	–	137 (16.3)	
60–64	–	169 (20.1)	
65–69	–	172 (20.5)	
70–74	–	141 (16.8)	
75–79	–	83 (9.8)	
80–84	–	31 (3.7)	
≥85	–	7 (0.8)	
**Gender, n (%)**			
Male	95 (59.7)	510 (60.6)	0.86
Female	64 (40.3)	331 (39.4)	
**Ethnic group, n (%)**			<0.001
Malay	91 (57.2)	341 (40.5)	
Chinese	62 (39.0)	468 (55.6)	<0.001
Indian	3 (1.9)	29 (3.5)	
Other	3 (1.9)	3 (0.4)	
**Family history of colorectal cancer, n (%)** Familial Adenomatous Polyposis Lynch Syndrome Sporadic	**n = 4 (2.5)** 2 1 1	**n = 18 (2.1)** 0 3 15	0.04
**Comorbidity, n (%)**			
Diabetes mellitus	13 (8.2)	107 (12.7)	
Hypertension	15 (9.4)	170 (20.2)	0.65
Dyslipidemia	5 (3.1)	63 (7.5)	
Ischemic heart disease	2 (1.3)	38 (4.5)	
**Treatment modality, n (%)** Surgery alone Surgery and Chemotherapy/Radiotherapy Surgery, Chemotherapy, and Radiotherapy Chemotherapy alone Chemotherapy and Radiotherapy Radiotherapy alone	**n = 134** 56 (41.8) 50 (37.3) 24 (17.9) 1 (0.7) 3 (2.2) 0	**n = 714** 347 (48.6) 233 (32.6) 118 (16.5) 9 (1.3) 5 (0.7) 2 (0.3)	0.56
Treatment modality not reported	25	127	
**Type of surgery, n (%)** Anterior resection Abdominoperineal resection Right hemicolectomy (plus extended) Left hemicolectomy (plus extended) Sigmoid colectomy Hartmann’s procedure Subtotal/total colectomy Panproctocolectomy Other	**n = 127** 40 (31.5) 18 (14.2) 18 (14.2) 15 (11.8) 1 (0.8) 7 (5.5) 3 (2.4) 3 (2.4) 12 (9.4)	**n = 703** 315 (44.8) 45 (6.4) 102 (14.5) 41 (5.8) 45 (6.4) 48 (6.8) 23 (3.3) 3 (0.4) 50 (7.1)	<0.001

### Treatment modalities received by CRC patients: EOCRC *vs.*≥50 years

There was a similar pattern regarding the treatments received by both age groups, with surgery alone being the most common treatment modality received by CRC patients (41.8% *vs.* 47.4% among EOCRC and those aged ≥50 years, respectively). The other treatments offered to the CRC patients are detailed in Table 2.

### Clinicopathological characteristics of CRC patients: EOCRC *vs.*≥50 years

A majority of the patients in both age groups had distal CRC on diagnosis (86% in EOCRC *vs.* 84% in those aged ≥50 years). Specifically, the rectum was the most common location of CRC (72.8% in EOCRC and 61.1% in those aged ≥50 years). The most common histological types of CRC were similar in both age groups: adenocarcinoma (87.4% *vs.* 93.6%) followed by mucinous adenocarcinoma (8.2% *vs.* 5.5%). When looking at the CRC staging, a higher proportion of patients with EOCRC were at an advanced stage at diagnosis (Dukes D: 19.9%) as compared to the patients aged ≥50 years (Dukes D: 10%) (Table 3).

**Table 3:** Clinicopathological characteristics of the two main examined age groups of colorectal cancer patients.

**Characteristics**	Age < 50 years, (total:159)	Age ≥50 years, (total:787)	*P* value
**Colon cancer, n (%)** Caecum Ascending colon Hepatic flexure Transverse colon Splenic flexure Descending colon Sigmoid Synchronous colon cancer	**n = 51 (35.7)** 9 (17.6) 8 (15.7) 0 3 (5.9) 3 (5.9) 10 (19.6) 14 (27.5) 4 (7.8)	**n = 301 (38.2)** 35 (11.6) 36 (11.9) 20 (6.6) 24 (7.9) 7 (2.3) 28 (9.3) 139 (46.2) 12 (4.0)	.034
**Rectal cancer, n (%)** Rectosigmoid Rectum Anus Synchronous colon + rectum cancer	**n = 92 (64.3)** 25 (27.1) 67 (72.8) 0 0	**n = 460 (58.4)** 171 (37.2) 281 (61.1) 1 (0.2) 7 (1.5)	0.14
**Cancer location, n (%)** Proximal CRC Distal CRC	20 (14.0) 119 (86.0)	122 (16.0) 639 (84.0)	0.63
Tumor location not reported	16	26	
**Histological type, n (%)** Adenocarcinoma Mucinous adenocarcinoma Signet ring cell carcinoma Squamous cell carcinoma Other	139 (87.4) 13 (8.2) 7 (4.4) 0 0	737 (93.6) 43 (5.5) 3 (0.4) 1 (0.1) 3 (0.4)	<0.001
**Histological grade, n (%)** Well differentiated Moderately differentiated Poorly differentiated Undifferentiated	**n = 115** 64 (55.7) 42 (36.5) 9 (7.8) 0	**n = 701** 381 (54.4) 286 (40.8) 31 (4.4) 3 (0.4)	0.36
Histological grade not reported	44	86	
**Dukes stage** A B C D	**n = 136** 7 (5.1) 38 (27.9) 64 (47.1) 27 (19.9)	**n = 758** 50 (6.6) 298 (39.3) 334 (44.1) 76 (10.0)	0.003
Dukes stage not reported	23	29	
**CEA at diagnosis** Not elevated (<5.0 ng/ml) Elevated (≥5.1ng/ml)	**n = 63** 34 (54.0) 29 (46.0)	**n = 360** 172 (47.8) 188 (52.2)	0.36
CEA level not reported	96	427	

## Discussion

**Figure 5:**
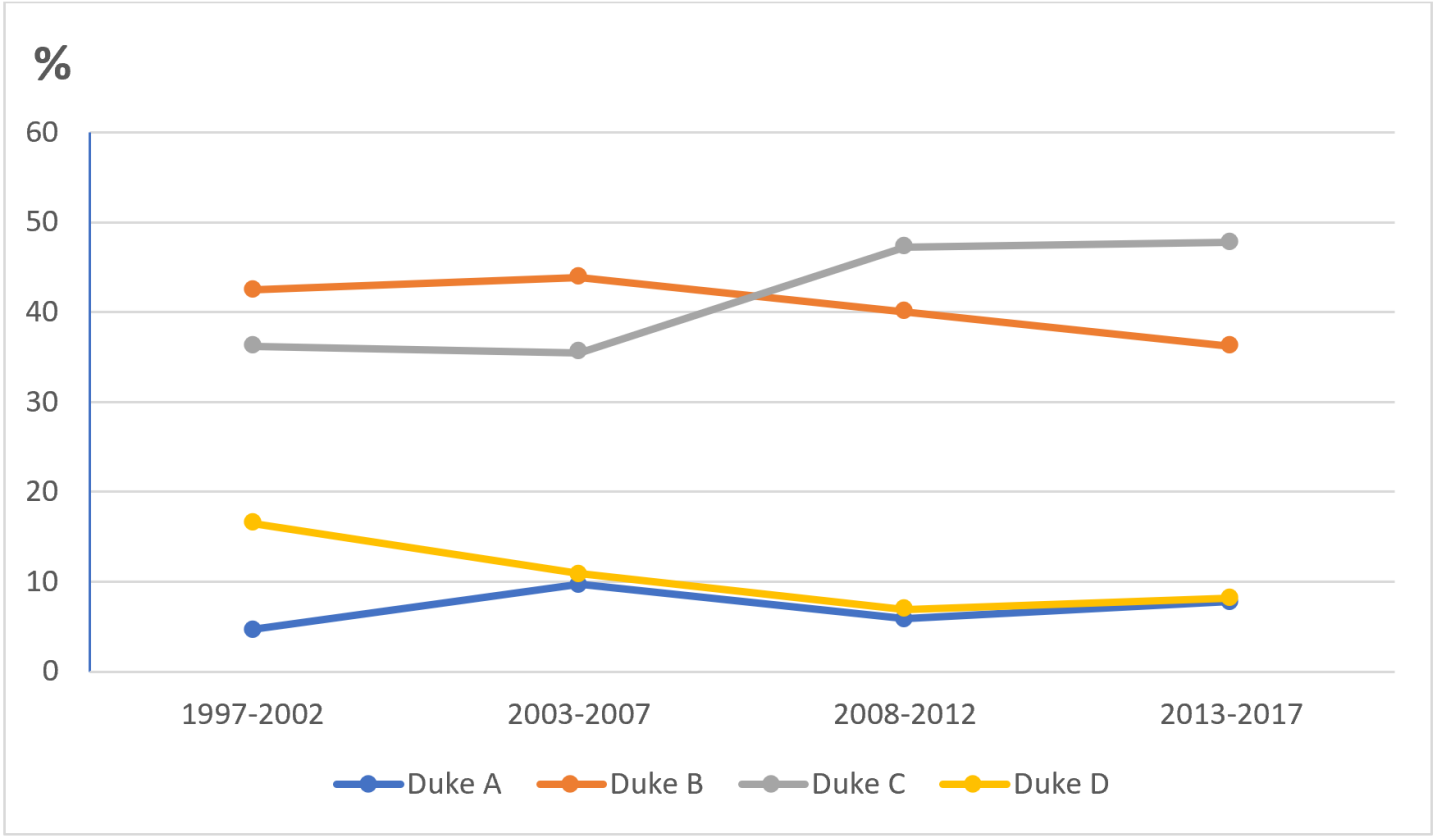
Time trend for proportion of CRC patients stratified by Dukes’ stage.

There was a substantial increase in the mean incidence of CRC at UKMMC during the 2008–2012 period, followed by a decreasing trend during the 2013–2017 period. This increment could be explained by changes in dietary patterns and sedentary lifestyles, partly due to improving socioeconomic status with a more westernized lifestyle being adapted (Center et al., 2009). A systematic review on diet and CRC risk in Asia showed that red meats, processed meats, preserved foods, and saturated fats have a positive association with CRC risk (Azeem et al., 2015). Furthermore, a case–control study in Malaysia revealed that high intake of red meat (OR = 6.52, 95% CI [1.93–2.04], *p* = 0.03) and lack of physical exercise are associated with CRC risk factors (Ramzi et al., 2014). In developed countries with established CRC screening programs, an initial increase in the CRC detection rate could be due to the increased screening uptake. However, this might not be the explanation for UKMMC’s CRC trend. In the past decade at UKMMC, although CRC screening was actively offered to average-risk patients, it was still quite selective, nonuniform, and opportunity-based. This was echoed in the time trend for CRC patients stratified by Dukes stage, where the number of cases at Dukes C continued to rise over time, while the numbers of cases at Dukes A and B remained low (Fig. 5). Although a majority of residents in semiurban areas in Malaysia were found to have good knowledge on CRC symptoms (67.3% for altered bowel habits and 63.4% for rectal bleeding), a significant proportion of them (22%) were not aware of any screening test for CRC (Naing et al., 2014). A survey by Hilmi, Hartono & Goh (2010), on the other hand, found that negative perceptions of CRC screening had prevented 62% of the respondents from willingly participating in CRC screening.

Ethnic disparities in CRC incidence are well documented in the literature. The latest nationwide CRC cohort (2012–2016) analysis revealed a higher incidence rate in those of Chinese ethnicity (male: 19.6 and female: 15.2 over 100,000) as compared to those of Malay ethnicity (male: 12.2 and female: 9.4) (Azizah et al., 2019). Data from a close neighboring country, from the Singapore Cancer Registry (2011–2015), showed a wide difference between the Chinese and Malay ethnic groups, with crude incidence rates for male CRC of 67.3 *vs.* 31.8, respectively (52.5 *vs.* 29.4, respectively, for female CRC) (National Registry of Disease Office, 2017). Our findings, however, revealed a reverse pattern, in which CRC incidences were higher in the Malay subgroup throughout the two decades, as compared to the Chinese subgroup. This reverse pattern was observed in our institution despite our catchment area being predominantly resided in by the Chinese community. One of the explanations, possibly due to different health-seeking behavior among the 3 major ethnic groups. A survey conducted in Kuala Lumpur (*n* = 991) revealed the Chinese was found to be less willing to undergo CRC screening compared to the Malay and Indian, independent of education level or knowledge of CRC and CRC screening (Hilmi, Hartono & Goh, 2010). Other study also has also shown that Chinese had the lowest uptake of medical services as compared to the other ethnicities (Krishnaswamy et al., 2009). In addition, our data were analyzed from a single center, rather than a population-based study, therefore this finding should be interpreted with caution.

Recently, data have been emerging on the increasing incidence of EOCRC. In Europe, on average, the CRC incidence rate increased by 4.9% per year from 2005 to 2016 in the age group 30–39 years and by 1.6% from 2004 to 2016 in the age group 40–49 years (Vuik et al., 2019). Similar patterns were shown for EOCRC in Asian countries (Sung et al., 2019). These global findings correlate with our data for the age groups 40–44 years and 45–49 years, but not for the age group 35–39 years. To date, no clinical risk factors have been identified, although changes in health behavior such as obesity, sedentary lifestyle, and environmental factors might contribute towards this increasing trend of CRC among young patients (Tan et al., 2019). In addition, EOCRC might carry a pathogenic germline variant in genes associated with predisposition to cancer (Venugopal & Stoffel, 2019). Microsatellite instability positivity was found in 14% of 93 cases referred for genetic testing (Sung et al., 2019). In this present study, unfortunately, most of the patients with EOCRC did not undergo genetic testing due to limited availability of genetic testing laboratory and more commonly, the financial issues. However, all patients did have a detailed, documented family history inquiry.

Worryingly, the incidence of EOCRC in Malaysia is starting to rise. Full commitment and collaboration from all relevant stakeholders, namely, health professionals from government and private sectors, government authorities, non-governmental organizations, media, and the public, are much needed in order to increase awareness of CRC and to promote preventive health-seeking behavior among the public. At the moment, the CRC screening strategy in Malaysia is selective opportunistic and therefore does not capture most of the average- and moderate-risk groups of CRC patients. There is an ongoing discussion on the implementation of population-based CRC screening in Malaysia. However, policymakers need to realize this increasing CRC trend, expedite the planning process, and make the long-overdue nationwide CRC screening in Malaysia a reality.

In our cohort, there was a higher proportion of CRC patients who were diagnosed at the later stages, especially young Malay adults. Multiple factors could contribute to the delay in diagnosis. An interesting survey conducted in Canada found that 26% of patients delayed seeking treatment because they did not consider their symptoms to be serious. Eighteen percent attributed the delay to the long waiting list for specialist review (Tomlinson et al., 2012). This survey highlighted the important patient and clinician factors for diagnosis delay. Therefore, ongoing education of the public and clinicians regarding signs and symptoms is needed, together with establishment of an efficient referral system for screening and suspected CRC cases. On the other hand, the reason for advanced and more aggressive disease in patients with EOCRC could be due to different biological behaviors of CRC between young and older patients. There was a higher incidence of mucin production and high microsatellite instability (MSI-H) in the younger CRC patients (Cheong et al., 2019). Despite this, younger patients have actually shown superior overall and cancer-specific survival rates as compared to patients with later-onset CRC (Rodriguez et al., 2018). This might be attributed to the less comorbidity and fewer complications from surgery and adjuvant chemotherapy/radiotherapy.

The rectum was the most common location for CRC among all colonic segments. This finding was in line with previous studies (Azmi, Zailani & Norashikin, 2007; Ghazali et al., 2010; Keum & Giovannucci, 2019). The proportion of rectal cancer was slightly higher in patients with EOCRC as compared to those with later-onset CRC. This has potential implications for screening criteria among the young population. Emphasis on any anorectal symptoms, such as tenesmus, anal pain, and rectal discharge, is paramount. Similarly, history of suspected external/internal hemorrhoids should be taken seriously, and patients should be subjected to colonoscopy or sigmoidoscopy at the very least.

One of the strengths of our study was employing a large number of CRC patients over a considerable period of time (20 years). Despite this, the present study did have some limitations. This was a retrospective–prospective cohort study; therefore, some of the required information was missing and unable to be retrieved. In addition, this was an urban, single-center study which would be prone to bias. The sample size for patients with EOCRC was also quite small; therefore, this study’s findings should be interpreted with caution in terms of their generalizability. However, we do feel that the obtained data are sufficient to reflect the clinico-epidemiology of CRC in Kuala Lumpur, the capital city of Malaysia, with a population of 1.8 million, and thus add to the current knowledge pertaining to CRC in Malaysia.

## Conclusions

The CRC incidence rate at UKMMC substantially increased at the start of the second examined decade (2008–2012) before declining towards the end of the study period (2013–2017). Upsettingly, although the total CRC incidence rate was downward-trending, this was not indicative of early detection of CRC patients. Moreover, CRC incidence among Malay patients aged less than 50 years was increasing, predominantly located at the rectal region, and mostly diagnosed at the later stages. It is expected that the incidence of CRC, especially in the younger population aged less than 50 years, will continue to rise for many years to come. All relevant stakeholders must continue to work on the management and prevention of CRC in Malaysia.

## Supplemental Information

10.7717/peerj.12425/supp-1 Supplemental Information 1 Raw data: Incidence

10.7717/peerj.12425/supp-2 Supplemental Information 2 Raw Data

## Additional Information and Declarations

### Data Availability

The following information was supplied regarding data availability: The data is available in the Supplemental Files.
