# Supplementary material for: Incidence and clinicopathological features of colorectal cancer among multi-ethnic patients in Kuala Lumpur, Malaysia: a hospital-based retrospective analysis over two decades
Source: PeerJ. 2021 Nov 8;9:e12425. doi: 10.7717/peerj.12425 (PMC8582301; doi:10.7717/peerj.12425)
Supplement: Supplemental Information 1 [file peerj-09-12425-s001.docx]

Table 1: ethnicity

| Year | Total CRC cases for each time period | | | | Kuala Lumpur Population (‘000) | | | | Cumulative Population (‘000) | | | | Mean Incidence  (100,000 person-year) | | | |
| --- | --- | --- | --- | --- | --- | --- | --- | --- | --- | --- | --- | --- | --- | --- | --- | --- |
|  | Malay | Chinese | Indian | Other | Malay | Chinese | Indian | Other | M | C | I | O | M | C | I | O |
| 1997 + 1998 | 63 | 79 | 2 | 3 | 551.4 | 555.2 | 144.5 | 16.5 | 2871.5 | 2875.1 | 743.5 | 86.2 | 2.19 | 2.75 | 0.27 | 3.48 |
| 1999 |  |  |  |  | 559.5 | 563.3 | 146.6 | 16.7 |  |  |  |  |  |  |  |  |
| 2000 |  |  |  |  | 572.7 | 576.6 | 149.6 | 17.6 |  |  |  |  |  |  |  |  |
| 2001 |  |  |  |  | 587.0 | 586.0 | 150.8 | 17.7 |  |  |  |  |  |  |  |  |
| 2002 |  |  |  |  | 600.9 | 594.0 | 152.0 | 17.7 |  |  |  |  |  |  |  |  |
| 2003 | 62 | 101 | 6 | 2 | 613.6 | 602.0 | 153.0 | 17.9 | 3194.8 | 3087.5 | 773.5 | 90.7 | 1.94 | 3.27 | 0.78 | 2.21 |
| 2004 |  |  |  |  | 626.3 | 609.6 | 153.9 | 18.0 |  |  |  |  |  |  |  |  |
| 2005 |  |  |  |  | 638.8 | 617.4 | 154.8 | 18.1 |  |  |  |  |  |  |  |  |
| 2006 |  |  |  |  | 651.5 | 625.3 | 155.5 | 18.3 |  |  |  |  |  |  |  |  |
| 2007 |  |  |  |  | 664.6 | 633.2 | 156.3 | 18.4 |  |  |  |  |  |  |  |  |
| 2008 | 138 | 197 | 13 | 7 | 677.7 | 641.2 | 156.9 | 18.6 | 3697.0 | 3461.6 | 834.9 | 85.7 | 3.73 | 5.69 | 1.56 | 8.17 |
| 2009 |  |  |  |  | 690.5 | 648.8 | 157.5 | 18.1 |  |  |  |  |  |  |  |  |
| 2010 |  |  |  |  | 770.4 | 716.8 | 172.5 | 15.1 |  |  |  |  |  |  |  |  |
| 2011 |  |  |  |  | 778.8 | 722.9 | 174.4 | 16.9 |  |  |  |  |  |  |  |  |
| 2012 |  |  |  |  | 779.6 | 731.9 | 173.6 | 17.0 |  |  |  |  |  |  |  |  |
| 2013 | 159 | 146 | 10 | 4 | 794.5 | 737.6 | 174.1 | 17.2 | 4100.0 | 3748.1 | 876.9 | 98.9 | 3.88 | 3.90 | 1.14 | 4.04 |
| 2014 |  |  |  |  | 804.4 | 740.1 | 173.7 | 19.1 |  |  |  |  |  |  |  |  |
| 2015 |  |  |  |  | 826.1 | 756.7 | 178.0 | 19.6 |  |  |  |  |  |  |  |  |
| 2016 |  |  |  |  | 835.8 | 757.0 | 175.4 | 21.5 |  |  |  |  |  |  |  |  |
| 2017 |  |  |  |  | 839.2 | 756.7 | 175.7 | 21.5 |  |  |  |  |  |  |  |  |
| unknown | 2 | 6 | 0 | 0 |  |  |  |  | - | | | | - | | | |

Table 2: gender

| Year | Total CRC cases for each time period | | Kuala Lumpur Population (by gender) (‘000) | | Cumulative Population (‘000) | | Mean Incidence  (100,000 person-year) | |
| --- | --- | --- | --- | --- | --- | --- | --- | --- |
|  | Male | Female | Male | Female | Male | Female | Male | Female |
| 1997 + 1998 | 79 | 67 | 693.4 | 574.2 | 3603.8 | 3286.2 | 2.19 | 2.04 |
| 1999 |  |  | 703.5 | 582.6 |  |  |  |  |
| 2000 |  |  | 720.2 | 695.8 |  |  |  |  |
| 2001 |  |  | 736.1 | 710.1 |  |  |  |  |
| 2002 |  |  | 750.6 | 723.5 |  |  |  |  |
| 2003 | 89 | 82 | 764.6 | 735.9 | 3958.5 | 3801.0 | 2.25 | 2.16 |
| 2004 |  |  | 778.1 | 748.3 |  |  |  |  |
| 2005 |  |  | 791.6 | 760.2 |  |  |  |  |
| 2006 |  |  | 805.3 | 772.2 |  |  |  |  |
| 2007 |  |  | 818.9 | 784.4 |  |  |  |  |
| 2008 | 232 | 124 | 832.4 | 796.5 | 4266.1 | 4085.4 | 5.44 | 3.04 |
| 2009 |  |  | 844.9 | 807.9 |  |  |  |  |
| 2010 |  |  | 855.9 | 818.8 |  |  |  |  |
| 2011 |  |  | 865.0 | 828.0 |  |  |  |  |
| 2012 |  |  | 867.9 | 834.2 |  |  |  |  |
| 2013 | 201 | 118 | 880.2 | 843.2 | 4515.0 | 4309.1 | 4.45 | 2.74 |
| 2014 |  |  | 884.1 | 853.3 |  |  |  |  |
| 2015 |  |  | 913.0 | 867.4 |  |  |  |  |
| 2016 |  |  | 918.3 | 871.4 |  |  |  |  |
| 2017 |  |  | 919.4 | 873.8 |  |  |  |  |
| unknown | 4 | 4 |  |  | - | | - | |
